# Preventative effect of *Astragalus flavescens* on hepatic fibrosis in rats and its mechanism of action

**DOI:** 10.3892/etm.2013.1232

**Published:** 2013-07-23

**Authors:** RONG LI, GUANGRONG DAI, MENGYUN ZHAO, YONGHONG ZHANG, LI HUI, XUELI ZHANG, BIN JIN

**Affiliations:** 1 Department of Gastroenterology, The First Hospital of Xi’an City, Xi’an, Shaanxi 710002;; 2 The Third Department of Internal Medicine, The Fifth Hospital of Xi’an City, Xi’an, Shaanxi 710082;; 3 Department of Gastroenterology, The Affiliated Hospital of Yan’an University, Yan’an, Shaanxi 716000, P.R. China

**Keywords:** *Astragalus flavescens*, hepatic fibrosis, prevention, mechanism

## Abstract

The aim of this study was to investigate the preventative effect of *Astragalus flavescens* on hepatic fibrosis in rats and its mechanism of action. A total of 60 rats were randomly divided into normal control, model control, high-dose treatment and low-dose treatment groups, and a hepatic fibrosis model was established. The high- and low-dose treatment groups were treated with 2 g/100 g and 0.5 g/100 g *Astragalus flavescens*, respectively, once a day. Eight weeks following the initiation of treatment, the liver specimens of the rats were stained and observed under a light microscope. Hepatic fibrosis indices, specifically, type III precollagen (PC III), type IV collagen (C IV), hyaluronic acid (HA) and laminin (LN), were detected. Furthermore, the expression and localization of the hepatic fibrosis-related factors transforming growth factor-β1 (TGF-β1), connective tissue growth factor (CTGF) and platelet-derived growth factor-BB (PDGF-BB) were determined. The serum levels of hepatic fibrosis indices, and the liver tissue levels of hepatic fibrosis-related factors and collagen surface density in the model control group and the high- and low-dose treatment groups were significantly higher compared with those of the normal control group (P<0.05). In addition, the values in the two treatment groups were significantly lower compared with those of the model control group (P<0.05). The present study demonstrated that *Astragalus flavescens* effectively prevents hepatic fibrosis in rats. A possible mechanism for this is that it may reduce the expression levels of TGF-β1, PDGF-BB and CTGF, thereby inhibiting the activation of hepatic stellate cells and specifically blocking the signal transduction pathway of hepatic fibrosis.

## Introduction

Hepatic fibrosis is a disease characterized by the abnormal hyperplasia of fibrous connective tissue in the liver, due to hepatocellular necrosis and inflammatory stimulation. It is the result of increased synthesis and decreased degradation of the extracellular matrix (ECM). Hepatic fibrosis is a common pathological process of all chronic liver diseases, and is the necessary prerequisite for liver cirrhosis (1). At present, it is mainly considered that the process of hepatic fibrosis is reversible, and its prevention and treatment is important for the prevention of liver cirrhosis (2). In recent years, studies concerned with the blockade or reversal of hepatic fibrosis have become an important topic in medicine worldwide (3-5). Due to increased investigation into the development of hepatic fibrosis (6-9), considerable progress has been made in the treatment of hepatic fibrosis with the emergence of novel synthetic drugs. However, the majority of these drugs are in clinical trials, with uncertain antifibrotic efficacy and significant side-effects.

At present, there are a number of single and compound prescriptions of traditional Chinese medicine that have demonstrated clear efficacy and marginal side-effects, and it has been suggested they may serve as potential treatments for hepatic fibrosis (10,11). *Astragalus flavescens,* a compound prescription of Chinese herbal medicine, contains Radix Astragali, Radix Scutellariae and Flavescent Sophora. *Astragalus flavescens* has been utilized in the treatment of chronic liver disease for >10 years and has shown curative effects. It has been clinically demonstrated that *Astragalus flavescens* is effective in receding jaundice, detumescence, retracting the spleen, reducing ascites, lowering transaminase activity, increasing serum albumin levels and improving the prothrombin time. Clinical studies have indicated that *Astragalus flavescens* is able to regulate the immunity and scavenge oxygen free radicals, as well as exhibiting anti-viral and -tumor effects. In addition, the prescription for *Astragalus flavescens* is low in cost and convenient to use; therefore, it has good developmental value. The present study investigated the preventative effects of *Astragalus flavescens* on liver fibrosis in rats and its mechanism of action. The study aimed to provide a reliable experimental basis for the further application of *Astragalus flavescens* in the treatment of hepatic fibrosis.

## Materials and methods

### Experimental groups

A total of 60 male Wistar rats (clean grade; average weight, 180 g), purchased from the Animal Experimental Center of the Fourth Military Medical University (Xi’an, China), were randomly divided into normal control, model control, high-dose treatment and low-dose treatment groups (n=15 rats per group). This study was carried out in strict accordance with the recommendations in the Guide for the Care and Use of Laboratory Animals of the National Institutes of Health (8th edition, 2011). The animal use protocol was reviewed and approved by the Institutional Animal Care and Use Committee (IACUC) of the First Hospital of Xi’an City (Xi’an, China). In the normal control group, a normal diet and water were freely available, 0.9% NaCl was administered to the rats by gavage daily and peanut oil was administered by subcutaneous injection at a dose of 0.5 ml/100 g on the first day and 0.3 ml/100 g once every 4 days thereafter. In the model control group, according to a modification of composite factor modeling methods (12,13), rats were subcutaneously injected with a mixture of 40% CCl_4_ and peanut oil at a dose of 0.5 ml/100 g on the first day and 0.3 ml/100 g once every 4 days thereafter. The rats were fed with freely available compound feed containing 79.5% pure flour, 20% lard and 0.5% cholesterol, and water was the only drink. In the high- and low-dose treatment groups, the modeling method was the same as that in the model control group; however, the rats were additionally treated with *Astragalus flavescens* (2 g crude drug/g powder; Xi’an Chinese Traditional Medicine Oral Tablet Factory, Xi’an, China) by gavage, once a day. The dosages were 2 g/100 g weight and 0.5 g/100 g weight, respectively. Eight weeks following the initiation of treatment, all rats were sacrificed and heart, blood and liver specimens were obtained.

### Hepatic fibrosis indices

The hepatic fibrosis indices, specifically, type III precollagen (PC III), type IV collagen (C IV), hyaluronic acid (HA) and laminin (LN), in the rat serum were detected by specific personnel in the isotope department using the radioimmunoassay method.

### Liver tissue specimen observation

Rat liver tissue specimens were stained with Masson’s trichrome (14,15), followed by observation under a light microscope. The collagen surface density in the liver tissue was calculated as follows: Collagen surface density (%) = (collagen area/viewed area) × 100.

### Hepatic fibrosis-related factors

Liver tissue paraffin sections were prepared and dewaxed, enzyme closure was performed using with 3% hydrogen peroxide and antigen retrieval with citrate buffer. After closing non-specific sites using non-immune goat serum, primary antibody (rabbit anti-rat TGF-β1 polyclonal antibody, rabbit anti-rat PDGF-BB polyclonal antibody and rabbit anti-rat CTGF polyclonal antibody; Wuhan Boster Biological Technology, Ltd., Wuhan, China) with 1:50 dilution using PBS was added, followed by incubation at 4°C overnight. After adding polymer enhancer and PBS washing, 50 *μ*l of horseradish peroxidase-labeled secondary antibody polymer was added by drops to each section, followed by incubation at 37°C for 30 min and 3 PBS washes. After coloration, counter-stain and mounting, the sections were observed in Q550CW image acquisition and analysis system (Leica Science Lab, Berlin, Germany). PBS replacing primary antibody was used as a blank, normal serum replacing secondary antibody was used as a negative control. No coloration was regarded as a negative result. TGF-β1, CTGF and PDGF were stained in the cytoplasm and cytomembrane. The brown or dark brown granular staining was defined as a positive result, and the staining significantly darker than background or no background staining referred to positively stained cells. Ten visual fields of each section were selected and the ratio of positive cell area to liver visual field area (*μ*m*^2^*/*μ*m*^2^*) was calculated. The broad-spectrum immunohistochemistry EliVision™ plus kit was provided by Fuzhou Maixin Biotechnology Development Co., Ltd. (Fuzhou, China).

### Statistical analysis

Data are expressed as the mean ± standard deviation. Statistical analysis was performed using SPSS software, version 11.5 (SPSS, Inc., Chicago, IL, USA). The Student’s t-test was used to analyze the differences between two groups, and single factor analysis of variance and Student-Newman-Keuls tests were conducted for comparisons among multiple groups. P<0.05 and P<0.01 were considered to indicate a statistically significant difference.

## Results

### Comparison of serum hepatic fibrosis indices

The serum levels of hepatic fibrosis indices in the model control and low- and high-dose treatment groups were significantly increased compared with those in the normal control group (P<0.05), and those in the two treatment groups were significantly lower than those in the model control group (P<0.05; Table I). The levels of PC III, C IV and LN in the high-dose treatment group were significantly lower than those in the low-dose treatment group (P<0.05); however, no significant difference was identified in the HA levels between the two treatment groups.

### Morphological changes

The collagen surface densities in the model control and low- and high-dose treatment groups were significantly increased compared with that in the normal control group (P<0.05; Table II). In addition, the collagen surface density in the two treatment groups was significantly lower than that in the model control group (P<0.05). Moreover, the collagen surface density in the high-dose treatment group was significantly lower than that in the low-dose treatment group (P<0.05).

### Expression of hepatic fibrosis-related factors

The expression levels of the hepatic fibrosis-related factors TGF-β1, CTGF and PDGF-BB in the different groups are shown in Table III. The expression levels of the three hepatic fibrosis-related factors in the model control group and the two treatment groups were significantly increased compared with those in the normal control group (P<0.05). Additionally, the expression levels of these factors in the two treatment groups were significantly lower than those in the model control group (P<0.05).

**Figure 1.**
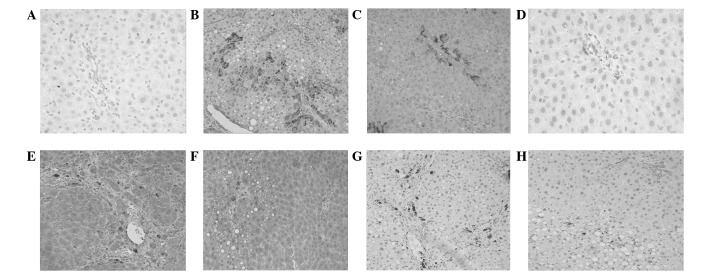
Immunohistochemical observations of the hepatic fibrosis related factors (A) transforming growth factor-β1 (TGF-β1) in the normal control group (magnification, ×400); (B) TGF-β1 in the model control group (magnification, ×200); (C) TGF-β1 in the high-dose treatment group (magnification, ×200); (D) connective tissue growth factor (CTGF) in the normal control group (magnification, ×400); (E) CTGF in the model control group (magnification, ×200); (F) CTGF in the high-dose treatment group (magnification, ×200); (G) platelet-derived growth factor-BB (PDGF-BB) in the model control group (magnification, ×400) and (H) PDGF-BB in the high-dose treatment group (magnification, ×200).

Expression of TGF-β1 was predominantly identified in the cytoplasm, and infrequently observed in the cytomembrane. The normal control group demonstrated weak expression of TGF-β1 in the cytoplasm of liver cells, with the distribution focused in the portal area and surrounding the central vein wall (Fig. 1A). TGF-β1 expression in the model control group was significantly increased compared with that of the normal control group, and the majority of the expression was observed in the portal area, central vein wall, proliferated fibrous septum, liver sinus wall, small bile duct cells, inflammatory cells and fatty and hepatic cells with steatosis and hydropic degeneration (Fig. 1B). The expression of TGF-β1 in the low- and high-dose treatment groups was significantly reduced compared with that of the model control group, and was mainly evident in the portal area, proliferated fibrous septum and inflammatory cells. Furthermore, the level of TGF-β1 expression in the high-dose treatment group was lower (Fig. 1C).

Expression of CTGF was observed in the cytoplasm and cytomembrane (as brown granules). In the normal control group, CTGF expression was negligible (Fig. 1D). By contrast, in the model control group, CTGF expression was significantly increased; the protein was predominantly identified in the spindle cells with processes and branches in the fibrosis portal area and fibrous septa [activated hepatic stellate cells (HSCs)], and inflammatory cells (Fig. 1E). The level of CTGF expression in the two treatment groups was significantly lower than that in the model control group, and that in the high-dose treatment group was the lowest (Fig. 1F).

Similarly to CTGF, PDGF-BB was expressed in the cytoplasm and cytomembrane. In the normal control group, no PDGF-BB expression was observed in hepatic cells and weak PDGF-BB expression was identified in the vascular wall, portal area and interstitial cells. In the model control group, the number of PDGF-BB-positive granules was significantly increased. Hyperplasia was most evident in the fibrous septa and portal area, with specific distribution in the infiltration area of inflammatory cells (Fig. 1G). The PDGF-BB expression level in the low- and high-dose treatment groups was significantly decreased compared with that of the model control group (Fig. 1H shows the expression in the high-dose treatment group).

## Discussion

Modern Chinese medicine considers liver blood stasis to be the main pathogenesis of hepatic fibrosis, of which the essence is fibrous tissue hyperplasia, degeneration and microcirculation disturbance. Therefore, activating blood and dissolving stasis is important for the treatment of hepatic fibrosis (16,17). *Astragalus flavescens* is proposed to be involved in activating blood and dissolving stasis, strengthening body resistance, supplementing qi, heat-clearing and detoxifying, removing dampness and receding jaundice. It may provide multi-channel, -level and -target prevention of and treatment for hepatic fibrosis.

Previous studies have demonstrated that, during the process of hepatic fibrosis, HSCs are activated and transformed into myofibroblast-like cells (18). This is important in the formation of hepatic fibrosis. Although there are a variety of cells involved in the induction of hepatic fibrosis, activated HSCs are the main cells that promote the deposition of a large quantity of ECM (19–22). The initiation and activation of HSCs are mediated by TGF-β1, which is an intermediary agent between HSC initiation and hepatic fibrosis (23), and is one of the most important factors determining hepatic fibrosis (24-26). During hepatic fibrosis, TGF-β1 initiates and maintains the activation of HSCs in a paracrine and autocrine manner, and regulates the cell proliferation. Therefore, it is able to promote collagen gene transcription and ECM proliferation, and inhibit the synthesis and secretion of proteolytic enzymes, thus resulting in a reduction in ECM degradation. Once hepatic fibrosis is initiated, it continues and the gradually increased fibrosis results in cirrhosis. Therefore, the inhibition of TGF-β1 production or the blocking of its biological activity may inhibit the activation of HSCs. This is considered to be one of the most promising methods of antifibrotic therapy (27). It has been proposed that TGF-β1 is also involved in normal physiological activities, such as immune suppression and the inhibition of cell proliferation (28,29). Therefore, when using drugs to inhibit TGF-β1 expression in antifibrotic therapy, controlled doses are necessary.

CTGF is a downstream response element of TGF-β1 and a central channel for the activation of HSCs (30). CTGF inhibitors are able to selectively block the fibrogenic effect of TGF-β1, and this role of CTGF is more likely to influence the occurrence of fibrosis (30). Therefore, CTGF inhibitors may be used for the effective prevention and treatment of hepatic fibrosis (31). The present study demonstrated that following treatment with *Astragalus flavescens*, the hyperplasia of collagen fibers in rats with hepatic fibrosis was significantly reduced, and the expression levels of TGF-β1 and CTGF in the fibrous septum were significantly inhibited. These results indicate that *Astragalus flavescens* is able to reduce the expression of TGF-β1 and CTGF, inhibit the activation and proliferation of HSCs, and prevent the continued amplification effect of cell activation. In addition, it may reduce the generation of CTGF, thus selectively blocking the fibrogenic channel of TGF-β1.

At present, it is considered that PDGF-BB is the most effective mitogenic factor for inducing HSC proliferation and the related signal transduction. PDGF-BB promotes the activation and division of HSCs and stimulates collagen synthesis (32–34). The current study identified that the localization of PDGF distribution was consistent with the sites at which HSC and collagen deposition are present. The expression levels of PDGF in the low- and high-dose treatment groups were significantly lower than that in the model control group. The results indicate that *Astragalus flavescens* inhibited the expression of PDGF and the proliferation and activation of HSCs, and may therefore prevent the formation of hepatic fibrosis.

## Figures and Tables

**Table I.** Comparisons of serum hepatic fibrosis indices in different groups.

Groups	No. of rats	Hepatic fibrosis indices (*μ*g/l)			
		PC III	C IV	HA	LN
Normal control	15	13.20±1.12	4.90±0.62	104.36±25.30	27.46±6.56
Model control	15	40.01±0.52^a^	20.56±0.23^a^	315.20±98.39^a^	70.11±10.02^a^
Low-dose treatment	15	34.20±0.82^a,b^	14.52±0.42^a,b^	185.20±18.21^a,b^	59.54±7.58^a,b^
High-dose treatment	15	19.56±0.98^a–c^	7.98±0.91^a–c^	137.25±19.89^a,b^	48.59±9.82^a–c^

Data are expressed as the mean ± standard deviation.

a P<0.05 compared with the normal control group;

b P<0.05 compared with the model control group and

c P<0.05 compared with the low-dose treatment group.

PC III, type III precollagen; C IV, type IV collagen; HA, hyaluronic acid; LN, laminin.

**Table II.** Comparisons of collagen surface density in different groups.

Group	No. of rats	Collagen surface density (%)
Normal control	15	4.83±2.78
Model control	15	24.31±3.55^a^
Low-dose treatment	15	18.02±3.64^a,b^
High-dose treatment	15	7.97±1.06^a–c^

a P<0.05 compared with the normal control group;

b P<0.05 compared with the model control group and

c P<0.05 compared with the low-dose treatment group.

**Table III.** Expression levels of hepatic fibrosis-related factors in different groups.

Group	No. of rats	Hepatic fibrosis-related factors (μm^2^/*μ*m^2^)		
		TGF-β1	CTGF	PDGF-BB
Normal control	15	0.10±0.02	0.03±0.01	0.05±0.02
Model control	15	0.61±0.05^a^	0.80±0.02^a^	0.61±0.06^a^
Low-dose treatment	15	0.30±0.08^a,b^	0.11±0.05^a,b^	0.41±0.10^a,b^
High-dose treatment	15	0.13±0.08^a,b^	0.05±0.09^a,b^	0.18±0.01^a,b^

Data are expressed as the mean ± standard deviation.

a P<0.05 compared with the normal control group;

b P<0.05 compared with the model control group.

TGF-β1, transforming growth factor-β1; CTGF, connective tissue growth factor; PDGF-BB, platelet-derived growth factor-BB.
